# Preoperative and Postoperative Salivary Bacterial Counts in Infants Undergoing Cardiac Surgery: A Prospective Observational Study

**DOI:** 10.7759/cureus.69269

**Published:** 2024-09-12

**Authors:** Hiromi Honda, Madoka Funahara, Kanako Nose, Megumi Aoki, Sakiko Soutome, Kenichi Yanagita, Atsuko Nakamichi

**Affiliations:** 1 School of Oral Health Sciences, Faculty of Dentistry, Kyushu Dental University, Kitakyushu, JPN; 2 Department of Pediatric Dentistry, Fukuoka Children's Hospital, Fukuoka, JPN; 3 Department of Oral Health, Nagasaki University Graduate School of Biomedical Sciences, Nagasaki, JPN

**Keywords:** bacterial load, cardiac surgery, infants, postoperative infection, saliva

## Abstract

Introduction: Postoperative pneumonia may develop in infants after cardiac surgery; however, only a few reports are available on perioperative oral bacteria in infants. This study aimed to examine preoperative and postoperative salivary bacterial counts in infants undergoing cardiac surgery.

Materials and methods: The number of bacteria in the saliva of 105 infants (average age: 20 months) who underwent surgery for congenital heart disease was determined by culturing before and after surgery. Factors associated with changes in the bacterial count were further examined. Patients received systemic antimicrobials for an average of four days immediately before surgery.

Results:Postoperative salivary bacterial counts were higher in older patients, who had erupting teeth and had longer surgical times. The average number of colonies before surgery was 10^4.53 ^CFU/mL; on the day after surgery, this number significantly decreased to 10^3.68^ CFU/mL. The rate of reduction was especially high in infants without tooth eruption. The total number of bacterial colonies in saliva decreased after surgery, most likely because of the use of systemically administered antibiotics, and the rate of decrease was particularly high in infants without tooth eruptions.

Conclusion: This study examined the preoperative and postoperative salivary bacterial counts in infants undergoing cardiac surgery. In the future, we would like to further examine bacterial flora and the effects of perioperative oral care. This study provides insights into the development of new strategies for preventing and treating surgical site infections and pneumonia in children.

## Introduction

Surgical site infections (SSI) and pneumonia are severe postoperative complications that prolong hospital stays, increase medical costs, decrease the quality of life of patients, and lead to mortality in some cases [1]. They occur in pediatric cardiovascular surgery at a rate of 1.7-8% [2]. Risk factors for postoperative infection in pediatric cardiovascular surgery include neonates or infants, preoperative hospital stay >48 hours, preoperative ventilatory management, and long operation time [3,4].

In adult surgery, the number of bacteria in saliva increases as oral and swallowing functions deteriorate postoperatively [5,6]. Particularly in patients who are intubated, oral bacterial counts increase significantly because of the lack of oral self-cleaning [7,8]. Possible causes of postoperative infection include direct exposure of the wound to salivary bacteria, aspiration of saliva-containing pathogenic microorganisms into the lungs, and hematogenous spread from oral infection sites. Thus, it is important to eliminate oral infections and establish good oral hygiene before surgery. Oral care before and after surgery for oral cancer [9], gastric cancer [10], esophageal cancer [10-13], lung cancer [14], colorectal cancer [10,15], and pancreatic cancer [16] reduces the incidence of postoperative pneumonia and SSI. However, little is known about the efficacy of oral care during pediatric cardiac surgery. We intend to determine in the future whether oral care decreases the frequency of postoperative complications in pediatric patients undergoing cardiac surgery. Prior to this, the purpose of this study was to determine the preoperative and postoperative salivary bacterial counts in infants undergoing cardiac surgery.

## Materials and methods

Study design

This was a prospective observational study conducted at Fukuoka Children's Hospital.

Setting

The study included patients who underwent open heart surgery for congenital heart disease at Fukuoka Children's Hospital between July 2022 and December 2023 and visited the Pediatric Dentistry Department for a preoperative oral examination. Informed consent for participation in the study was obtained from the parents and guardians of all patients.

Patient demographics

The study included pediatric patients who were scheduled for open heart surgery for congenital heart disease. Relevant factors from the patient's medical records were examined, including sex, age (in months), body mass index, and tooth eruption.

Inclusion and exclusion criteria

Inclusion Criteria

Included were infants undergoing open heart surgery for congenital heart disease whose parents agreed to participate in the study.

Exclusion Criteria

Patients with an iodine allergy or those for whom the attending physician deemed oral care intervention impossible were excluded.

Data collection

The following factors were examined from the patient's medical records: sex, age (in months), body mass index, tooth eruption, serum albumin level, operation time, blood loss, and respiratory management after surgery (no management, high-flow nasal cannula (HFNC), nasal directional positive airway pressure (n-DPAP), or nasal continuous positive airway pressure (n-CPAP)). Oral wetness was measured using a dental mirror [17], and a score of 1 was classified as xerostomia (−) and a score of 1-2 as xerostomia (+).

Collecting saliva samples and bacterial culture

Saliva samples were collected before and on the day after surgery. The swab tip was inserted under the tongue using a Transwab ENT (Medical Wire & Equipment Co. Ltd., London, UK) for five seconds to allow saliva to enter the swab tip for collection. The tip of the swab was then suspended in 200 µL of saline for 20 seconds, and the suspended saline was used for culturing bacteria. Poremedia BHI agar (Eiken Chemical Co. Ltd., Tokyo, Japan) was used as the culture medium. The medium was incubated at 37°C for 48 hours, and the number of colonies was counted directly.

Statistical analysis

All statistical analyses were performed using the IBM SPSS Statistics for Windows, V. 26.0 (IBM Corp., Armonk, NY, USA). The relationship between factors associated with the number of bacteria in saliva before surgery, factors associated with the number of bacteria in saliva after surgery, and factors associated with the postoperative decrease rate were analyzed using one-way ANOVA for categorical data and Spearman's rank correlation coefficient for continuous data. Statistical significance was defined as a two-tailed p-value of <0.05. Factors associated with the number of bacteria in saliva after surgery and the postoperative decrease rate were analyzed using multivariate analysis. A multiple regression analysis was performed, including factors found to be significant in the univariate analysis. Statistical significance was defined as a two-tailed p-value of <0.05.

Ethical approval

This study was conducted in March 2022 in the Department of Oral Health Sciences, School of Dentistry, Kyushu Dental University, Kitakyushu, Japan. All procedures and materials were approved by the Ethics Committee of Kyushu Dental University (approval number: 21-40). Verbal informed consent was obtained from the parents or guardians of each participant before the study. The study protocol conformed to the ethical guidelines of the Declaration of Helsinki and the Ethical Guidelines for Medical and Health Research Involving Human Subjects of the Ministry of Health, Labor, and Welfare of Japan.

## Results

Patient characteristics

Participants included 105 infants (46 males and 59 females) with an average age of 20 months. Overall, 45 patients did not experience a tooth eruption. Dry mouth was not observed preoperatively in most of the patients. The mean operative time was 379 minutes, the mean blood loss was 107 g, and the patients received intravenous antimicrobials for an average of four days postoperatively (Table 1).

**Table 1:** Patient characteristics of 105 infants HFNC: high-flow nasal cannula; n-DPAP: nasal directional positive airway pressure; n-CPAP: nasal continuous positive airway pressure; SD: standard deviation

Variable		Number of patients/mean±SD
Sex	Male	46
	Female	59
Age		20.0±21.0
Body mass index		15.4±1.46
Tooth eruption	(−)	45
	(+)	60
Oral wetness (before surgery)	(−)	103
	(+)	2
Oral wetness (after surgery)	(−)	35
	(+)	70
Albumin	g/dL	4.52±0.338
Operation time	min	379±149
Blood loss	g×10^2^	1.07±2.49
Respiratory management	No management	28
	HFNC	68
	n-DPAP/n-CPAP	2
	Tracheotomy	7
Antibacterial drug delivery	Days	4.0±1.7
Total		105

Number of bacteria in saliva before surgery

The mean number of colonies in preoperative saliva samples was 10^4.53^ CFU/mL. The number of bacteria was the dependent variable, and the factors affecting it were examined. However, no factors related to the preoperative bacterial count were found (Table 2).

**Table 2:** Factors associated with the number of bacteria in saliva before surgery ^§^One-way ANOVA; ^†^Spearman's rank correlation coefficient; *p<0.05; **p<0.01. A univariate analysis was conducted to identify factors associated with the number of bacteria in saliva before surgery. No significant factors influencing the number of bacteria in saliva were found among the factors examined.

Variable (categorical variable)		Number of patients	Number of total bacteria (logarithm) before surgery	P-value^§^
Sex	Male	59	4.54±1.14	0.885
	Female	46	4.51±0.756	
Tooth eruption	(−)	45	4.61±0.844	0.450
	(+)	60	4.47±1.09	
Oral wetness (before surgery)	(−)	103	4.53±0.997	0.798
	(+)	2	4.35±0.365	
Variable (continuous variable)			Spearman's correlation coefficient	P-value^†^
Age	Weeks		0.089	0.369
Body mass index			-0.083	0.401
Albumin	g/dL		0.110	0.263

Postoperative salivary bacterial counts and factors associated with changes from preoperative counts

Patients received systemic antimicrobials for an average of four days postoperatively. The mean colony count of all postoperative saliva samples was 10^3.68^ CFU/mL, which was significantly lower than that of preoperative samples. In the univariate analysis, postoperative bacterial counts were significantly higher in patients who were older (p<0.001), had erupted teeth (p<0.001), and had prolonged surgical times (p=0.038) (Table 3). The multivariate analysis showed that the number of postoperative salivary colonies was significantly higher in older patients (p=0.028) and tended to be higher in patients with prolonged surgical times (p=0.086) (Table 4).

**Table 3:** Factors associated with the number of bacteria in saliva after surgery ^§^One-way ANOVA; ^†^Spearman's rank correlation coefficient; *p<0.05; **p<0.01. A univariate analysis was conducted to identify factors associated with the number of bacteria in saliva after surgery. Significant differences were found between "tooth eruption," "age," "operation time," and the number of bacteria in saliva after surgery. HFNC: high-flow nasal cannula; n-DPAP: nasal directional positive airway pressure; n-CPAP: nasal continuous positive airway pressure

Categorical variable		Number of patients	Number of total bacteria (logarithm) after surgery	P-value^§^
Sex	Male	59	3.56±1.25	0.302
	Female	46	3.83±1.44	
Tooth eruption	(−)	45	3.17±1.40	**<0.001
	(+)	60	4.06±1.21	
Oral wetness (before surgery)	(−)	103	3.66±1.36	0.252
	(+)	2	4.77±0.723	
Oral wetness (after surgery)	(−)	35	3.59±1.23	0.640
	(+)	70	3.72±1.43	
Respiratory management	No management	28	3.70±1.23	0.743
	HFNC	68	3.68±1.45	
	n-DPAP/n-CPAP	2	2.67±1.28	
	Tracheotomy	7	3.89±1.08	
Continuous variable			Spearman's correlation coefficient	P-value^†^
Age	Months		0.432	**<0.001
Body mass index			-0.004	0.970
Albumin	g/dL		0.012	0.901
Operation time	min		0.203	*0.038
Blood loss	g×10^2^		0.106	0.282

**Table 4:** Factors associated with the number of bacteria in saliva after surgery A multivariate analysis was conducted to identify factors associated with the number of bacteria in saliva after surgery. Significant differences were observed in the factors "age" (*p<0.05).

Variable	Unstandardized coefficient		Standardized coefficient	95% confidence interval		P-value
	B	SE	β	Lower	Upper	
Age (in months)	0.02	0.01	0.255	0.002	0.031	*0.028
Tooth eruption	0.38	0.31	0.140	-0.233	1.002	0.220
Operation time (min)	0	0	0.160	0	0.003	0.086

Considering the logarithm of 100 for the preoperative bacterial count, the postoperative bacterial count decreased by an average of -13.8%. When examining the factors associated with the rate of increase as the dependent variable, univariate analysis showed that the degree of decrease was significantly greater in older patients (p<0.001) and infants with erupting teeth (p=0.003) (Table 5). In the multivariate analysis, tooth eruption was identified as an independent and significant factor (p=0.023) associated with a large postoperative bacterial count reduction rate, whereas age showed no significant association (p=0.816) (Table 6).

**Table 5:** Factors associated with the rate of postoperative decline ^§^One-way ANOVA; ^†^Spearman's rank correlation coefficient; *p<0.05; **p<0.01. A univariate analysis was conducted to identify factors associated with the increased rate in the number of bacteria in saliva before and after surgery. Significant differences were observed in "tooth eruption" and "age."

Categorical variable		Number of patients	Postoperative decrease rate	P-value^§^
Sex	Male	59	-14.6±53.5	0.827
	Female	46	-12.7±33.9	
Tooth eruption	(−)	45	-29.1±34.8	*0.003
	(+)	60	-2.28±49.8	
Oral wetness (before surgery)	(−)	103	-14.2±46.1	0.474
	(+)	2	9.35±7.42	
Oral wetness (after surgery)	(−)	35	-17.8±38.5	0.530
	(+)	70	-11.8±49.2	
Respiratory management	No management	28	-11.3±41.0	0.430
	HFNC	68	-16.2±48.7	
	n-DPAP/n-CPAP	2	-43.4±33.9	
	Tracheotomy	7	8.69±33.3	
Continuous variable			Spearman's correlation coefficient	P-value^†^
Age	Weeks		0.37	**<0.001
Body mass index			0.028	0.775
Albumin	g/dL		-0.018	0.853
Operation time	min		0.179	0.068
Blood loss	g×10^2^		0.119	0.226

**Table 6:** Factors associated with the postoperative decrease rate A multivariate analysis was conducted to identify factors associated with the increased rate in the number of bacteria in saliva before and after surgery. "Tooth eruption" was identified as an independent factor associated with the decrease rate in the number of bacteria in saliva after surgery (*p<0.05).

Variable	Unstandardized coefficient		Standardized coefficient	95% confidence interval		P-value
	B	SE	β	Lower	Upper	
Age	0.06	0.259	0.028	-0.453	0.574	0.816
Tooth eruption	25.272	10.94	0.274	3.569	46.98	*0.023

## Discussion

This study revealed that the number of bacterial colonies in infants undergoing cardiac surgery decreased postoperatively compared to preoperatively, presumably because of the effects of the systemic administration of antimicrobial agents. However, the decrease in colony counts was suppressed in older children with erupting teeth who underwent prolonged surgical time, and tooth eruption was an independent factor that was significantly associated with a lower reduction rate.

Various complications can develop after cardiac surgery in infants, of which pneumonia is the most common. Yu et al. reported that of 93 infants who were intubated after cardiac surgery, 14 (15%) developed postoperative pneumonia [18]. Jácomo et al. reported nosocomial pneumonia in 44 (27.5%) infants and ventilator-associated pneumonia (VAP) in 27 (16.9%) of 160 infants who underwent cardiac surgery [19]. Roeleveld et al. reported that out of 125 infants who underwent cardiac surgery and were intubated for >24 hours postoperatively, 18 (14.4%) developed pneumonia and 11 (8.8%) developed VAP [20]. Although preventing pneumonia after cardiac surgery in infants is important, only a few studies have investigated the number of bacteria in the saliva of infants during the perioperative period.

The oral cavity of the fetus is sterile; however, after birth, the infant is continuously exposed to microbial invasion through contact with humans and the environment, and the development of endogenous oral microflora begins. For bacteria, only the mucosal surfaces are available in the oral cavity of infants during the first few months of life, which limits the number of bacterial species that can colonize. However, the number of bacteria and bacterial flora change drastically when teeth erupt [21]. The number of bacteria in saliva increases following highly invasive surgery in adults owing to decreased oral self-cleaning as well as postoperative oral and swallowing dysfunction [6]. In the present study, the degree of xerostomia in infants increased in the postoperative period compared to that in the preoperative period, suggesting a decline in oral function; however, the number of bacteria in saliva was significantly lower in the postoperative period than in the adult period. One possible reason for this is the effect of the systemic administration of antimicrobial agents. We believe that systemically administered antimicrobial agents effectively reduce the number of bacteria in saliva. Furthermore, the number of bacteria in saliva is considerably lower in infants than in adults, and infants have fewer plaques and periodontal pockets, less biofilm formation, and fewer anaerobic bacteria than adults because of a lack of or fewer erupting teeth. This study showed no difference in the preoperative salivary bacterial counts before and after tooth eruption; however, the fact that the rate of decrease was greater in edentulous infants after surgery supports these hypotheses.

The outcome of the current study was not the development of postoperative pneumonia but the number of bacteria in saliva. Pneumonia is caused by a combination of the aspiration of salivary bacteria and decreased immunity. Preoperatively, the risk of pneumonia is low because aspiration does not usually occur, regardless of the number or type of bacteria in saliva. In contrast, the risk of pneumonia is higher after surgery because the patient is intubated and aspiration occurs despite a slight decrease in salivary bacterial counts due to the effects of systemic antibacterial drugs. Therefore, oral care to decrease the number of bacteria in saliva is necessary to prevent pneumonia. Although this study is preliminary and limited to a survey of salivary bacterial counts, we plan to investigate whether oral care interventions can further reduce these counts.

This study has some limitations. Although we found that antimicrobial treatment reduced the total number of bacteria in postoperative saliva, especially in infants before tooth eruption, we did not examine the bacterial species, and it is unclear whether the number of bacteria that can cause pneumonia was reduced. However, we believe that this study is significant because it is the first to examine preoperative and postoperative bacterial counts in infants undergoing cardiac surgery. Nevertheless, further studies are needed to examine the bacterial flora to ascertain whether perioperative oral care is as effective in infants as it is in adults and to determine the methods of oral care that should be implemented.

## Conclusions

In this study, the postoperative salivary bacterial counts were higher among older patients who had erupting teeth and experienced longer surgical times; however, the postoperative bacterial counts were often lower than the preoperative values. The rate of decrease was particularly high in the group of infants who did not have tooth eruption, possibly because of the effect of systemically administered antimicrobial agents. In a future study, we aim to examine the effects of oral care on the bacterial flora. After surgical intervention in infants, the bacterial count in saliva decreases compared to preoperative levels; however, the onset of VAP in neonates and infants has not been eradicated. Consequently, it is imperative to emphasize that oral care should focus on postoperative oral bacterial count.
